# Variation within laminae: Semi‐automated methods for quantifying leaf venation using phenoVein

**DOI:** 10.1002/aps3.11346

**Published:** 2020-05-11

**Authors:** Eastyn L. Newsome, Grace L. Brock, Jared Lutz, Robert L. Baker

**Affiliations:** ^1^ Department of Biology Miami University 700 E High Street Oxford Ohio 45056 USA

**Keywords:** areole area, *Brassica rapa*, leaf venation, phenoVein, venation density, venation variation

## Abstract

**Premise:**

Physiological processes may vary within leaf laminae; however, the accompanying heterogeneity in leaf venation is rarely investigated because its quantification can be time consuming. Here we introduce accelerated protocols using existing software to increase sample throughput and ask whether laminae venation varies among three crop types and four subspecies of *Brassica rapa*.

**Methods:**

FAA (formaldehyde, glacial acetic acid, and ethanol)‐fixed samples were stored in ethanol. Without performing any additional clearing or staining, we tested two methods of image acquisition at three locations along the proximal‐distal axis of the laminae and estimated the patterns of venation using the program phenoVein. We developed and made available an R script to handle the phenoVein output and then analyzed our data using linear mixed‐effects models.

**Results:**

Beyond fixation and storage, staining and clearing are not necessary to estimate leaf venation using phenoVein if the images are acquired using a stereomicroscope. All estimates of venation required some manual adjustment. We found a significant effect of location within the laminae for all aspects of venation.

**Discussion:**

By removing the clearing and staining steps and utilizing the semi‐automated program phenoVein, we quickly and cheaply acquired leaf venation data. Venation may be an important target for crop breeding efforts, particularly if intralaminar variation correlates with variation in physiological processes, which remains an open question.

The vasculature of leaves is essential for their structural support, the transport of photosynthates from the leaves to the rest of the plant, and for supplying water and minerals to the leaves (Raven et al., 2005; Sack and Scoffoni, 2013). Leaf venation is variable among species and, because of the potential yet poorly understood relationships between leaf venation and physiological function, it has been characterized as an important target of evolutionary selection (Roth‐Nebelsick et al., 2001). Leaf venation is also implicated in influencing ecosystem and agricultural productivity (Sack and Scoffoni, 2013). As human population growth puts increased demands on freshwater supplies and agricultural systems, quantifying leaf venation patterns may reveal potential new avenues for breeding water‐use efficient plants (Feldman et al., 2014; Mathan et al., 2016); however, relatively few studies have quantified venation, in part because collecting these data can be time consuming. To address this problem, we couple a fast, easy method of preparing and imaging leaves with existing semi‐automated leaf venation detection software, and we introduce a script to efficiently combine and reformat the data for downstream analyses. The increased speed of data acquisition allowed us to examine intralaminar variation in leaf venation.

Perhaps the most critical step for any image analysis platform is image acquisition. Poor‐quality images result in poor‐quality data. Acquiring high‐quality images often necessitates time‐ and labor‐intensive clearing, staining, mounting, and imaging protocols (Vasco et al., 2014). We explored a number of protocols and determined that minimal sample preparation is necessary to successfully estimate leaf venation patterns.

Once images are acquired, the data representing leaf venation patterns must be extracted from the images. Manually tracing leaf veins is possible, but it can be exceedingly tedious and labor intensive. Numerous programs are available for quantifying leaf venation (see https://www.quantitative-plant.org for some examples). We opted to use the program phenoVein because it is a free, well‐supported, intuitive tool with open‐source code (Bühler et al., 2015). Additionally, phenoVein is available offline and offers automated vein segmentation. One particularly useful aspect of phenoVein is the ability to manually correct the estimated venation networks. Problematically, the phenoVein file output format is not easily analyzed using standard statistical tools; therefore, we developed and make available here an R script (R Core Team, 2019) to concatenate multiple phenoVein output files, extract relevant data, and organize the information into a rectangular dataframe that is easily imported into any number of statistical analysis platforms.

The physiological function and structural properties of the leaf lamina may vary along the proximal‐distal axis. Previous work has described spatial variation within leaf lamina in traits such as stomatal conductance (Nardini et al., 2008; Ocheltree et al., 2012), stomatal density (Wang and Clarke, 1993), stomatal size (Weyers and Lawson, 1997), and the quantum yields of photosystem II (Fv/Fm; Enríquez et al., 2002); however, few studies have examined the heterogeneity in leaf venation within a single leaf. Some exceptions include particularly large leaves such as *Musa balbisiana* Colla (wild banana) and *Alocasia macrorhiza* Schott (giant taro), in which there was no variation in vein density across the lengths of the laminae (Li et al., 2013; Li and Cao, 2014). In contrast, *Nicotiana tabacum* L. (tobacco) leaves exhibit significantly greater vein density near the leaf apex compared to the base (Nardini et al., 2008). Whether the patterns of leaf venation vary along the proximal‐distal axis of *Brassica rapa* L. laminae remains an open question.

Here, we present a set of inexpensive, fast, and easy protocols for preparing and imaging leaves. We report on quantifying leaf venation patterns using phenoVein in diverse cultivars of *B. rapa* and provide estimates for computational times, as well as providing a freely available R script to facilitate the downstream data analysis of phenoVein output (R Core Team, 2019). We demonstrate the use of this set of tools by quantifying aspects of leaf venation at the base, middle, and apex of the leaf laminae from the crop species *B. rapa*, and ask whether there are differences in the patterns of venation across the proximal‐distal axis of the leaves.

## Methods

### Species description

*Brassica rapa* (Brassicaceae) is a herbaceous annual to biannual plant that was first domesticated in Eurasia (Kokichi and Shyam, 1984). Artificial selection has resulted in three morphologically distinct and genetically monophyletic crop types: vegetable turnips (subsp. *rapa*; VT), oil seeds (subsp. *oleifera* (DC.) Metzg. and subsp. *trilocularis* (Roxb.) Hanelt; OS), and Chinese cabbages (subsp. *pekinensis* (Lour.) Hanelt; CC; Qi et al., 2017).

### Study design, plant growth, and tissue collection

As part of a larger, previously published experiment (Baker et al., 2017), we obtained seeds of multiple genotypes (accessions) of each of the three crop types from the USDA Germplasm Information Network (GRIN), Ames, Iowa, USA, and the Centre for Genetic Resources (CGN) at Wageningen University & Research, The Netherlands (Table 1). Five separate blocks were sown, each containing one plant from each genotype, and grown in a single greenhouse room, with the plants spaced to minimize self‐shading. The actual number of plants per accession departed from five due to poor germination rates (Table 1).

**Table 1:** *Brassica rapa* plant material examined in the present study.

Accession ID	Crop type^a^	Subspecies	Germplasm source^b^	Sample size
Ames 2795	OS	*oleifera*	GRIN	2
CGN06709	VT	*rapa*	CGN	3
CGN06710	VT	*rapa*	CGN	3
CGN06711	VT	*rapa*	CGN	1
CGN06813	CC	*pekinensis*	CGN	3
CGN07143	CC	*pekinensis*	CGN	3
PI459016	OS	*trilocularis*	GRIN	3
PI459018	OS	*trilocularis*	GRIN	2
PI459020	OS	*trilocularis*	GRIN	3

a OS = oil seed; VT = vegetable turnip; CC = Chinese cabbage.

b GRIN = USDA Germplasm Information Network in Ames, Iowa, USA; CGN = Centre for Genetic Resources (CGN) at Wageningen University & Research, The Netherlands.

The third epicotylar leaf was collected from the leaf base within 36 h of fully expanding, as described by Baker et al. (2017). For the leaves used in this study, the leaf area (base plus petiole plus lamina) ranged from 8.16 cm^2^ to 110.39 cm^2^ (Baker et al., 2017). The leaves were fixed for 24 h in FAA (1 : 1 : 8 ratios of formaldehyde, glacial acetic acid, and ethanol by volume) and stored in 70% ethanol.

### Tissue preparation and image acquisition

No tissue preparation beyond fixation and storage (such as clearing or staining) was performed, with the exception of the occasional additional ethanol wash to keep leaves hydrated during imaging or to refill storage tubes as the ethanol was used or evaporated.

Images were acquired from the apex, middle, and base of each leaf. The main vein was avoided as much as possible, and the other major veins were avoided where possible. We also avoided damaged areas or folds in leaves as much as possible. We tested two image acquisition methods: using a digital single lens reflex (SLR) camera with a macro lens and using stereomicroscopes mounted with digital cameras.

#### Digital SLR camera

Images were captured with a Canon EOS Rebel T3i camera (Canon Inc., Tokyo, Japan) with a Canon EF‐S 60‐mm f/2.8 macro lens attached to a copy stand to maintain a consistent focal distance. LED light was transmitted from one of the light panels that shipped with the copy stand, which consisted of 96 LEDs with a color rendering index (CRI) of 97 emitting 5213 lux at 30.5 cm (Smith‐Victor, Bartlett, Illinois, USA). A piece of A4 office paper was used as a light diffuser, and was placed approximately 1 cm (the height of a Petri dish lid) from the light panel. Each leaf was placed in a large glass Petri dish and hydrated with 70% ethanol. The best images were acquired with an f/4 aperture, a shutter speed of 1/320, and ISO of 100 in RAW format (.CR2). The raw image files were converted to .tif files prior to being imported into phenoVein.

#### Stereomicroscopes

We used one of two different stereomicroscopes, a Leica M125 C (Leica Microsystems, Wetzlar, Germany) with a Leica MC170 HD camera or an Olympus SZX12 (Olympus, Tokyo, Japan) with a Nikon D300 camera (Nikon, Tokyo, Japan), to image leaves under transmitted light. We assumed image quality from each microscope was comparable. The leaves were kept hydrated throughout the imaging process using 70% ethanol. We imaged each leaf three times: once at the base, middle, and apex of the lamina using 10× or 12.5× magnification.

### phenoVein workflow

We loaded each image into phenoVein (version 1.0; Bühler et al., 2015), which is a software package within the free MeVisLab SDK suite (version 2.8.2) and is available for Windows, MacOS, and Linux operating systems at https://www.mevislab.de/ (accessed 6 April 2020). Based on a visual inspection, either the R or G color channels gave the best contrast and resolution; therefore, we chose channels on an image‐by‐image basis. We manually differentiated background from the leaf, which was important because the leaves did not always fill the entire field of view. Often, images included undesired features such as tears, folds, or damaged tissue. Within phenoVein, we masked these areas from subsequent image analyses. Next, we adjusted the upper and lower sigma values to a range generally falling within 12–26 (following Bühler et al., 2015), and then allowed phenoVein to estimate a network skeleton representing the vasculature system.

phenoVein superimposes the estimated network skeleton onto the original image, allowing for an easy visual confirmation of the estimated network. We adjusted the minimum vein ending setting to improve the match between the estimated network skeleton and the veins in the image. We also performed manual editing based on a visual inspection, which entailed inserting and deleting vein segments as needed. Images with good contrast between the veins and the mesophyll tissue required little manual correction, while images with poor contrast required substantial manual corrections. One common problem requiring manual correction was that phenoVein often identified larger veins as two parallel veins rather than a single wide vein. In these cases, we located the vein segment at the midpoint (or center) of the wide vein. Once we achieved a satisfactory match between the estimated vein network and the visually apparent veins in the image, we ran the phenoVein analysis using a standard desktop workstation with a single core i7 3.6‐Ghz processor and 8 Gb of RAM running Windows 10 (Microsoft Corporation, Redmond, Washington, USA). phenoVein generates several output files, including a comma‐separated values file (.csv) containing the data extracted from the image.

### Data management and analysis

To organize, process, and prepare the data generated using phenoVein for statistical analyses, we constructed a custom script for the R statistical computing environment (version 3.6.1; R Core Team, 2019). Included in the output files for phenoVein are all of the data generated for each image in a single .csv file; however, the data are formatted such that they are not easily analyzed using standard statistical tools. We developed an R script that (1) compiles data from multiple phenoVein .csv output files, (2) reformats the phenoVein output into a rectangular dataframe to facilitate the downstream data analysis, and (3) saves this new dataframe as a separate .csv file that can be easily imported into and analyzed using any number of statistical software environments (available at https://github.com/rlbaker5/AppsInPlantSci_phenoVein). Because this R script relies on regular expressions, maintaining consistent file names for both the original images as well as the phenoVein output is crucial.

While reshaping the phenoVein output, we chose not to include some data, but the user can easily edit our script to include these data if appropriate. For instance, phenoVein scores all unconnected areoles as a single large areole (Bühler et al., 2015). This may be appropriate when imaging whole leaves, such as the *Arabidopsis thaliana* (L.) Heynh. leaf described by Bühler et al. (2015), for which the leaf margin defines a biologically relevant and contiguous areole. However, in our study of much larger leaves, the large “edge” areole identified by phenoVein is often defined by the field of view and is therefore not biologically relevant. The R script developed here contains an option to remove the composite “edge” areole when working with larger leaves. We also opted not to use the vein width data in our analyses for the following reasons: (1) our vein widths varied greatly within a sample, (2) phenoVein occasionally called a single large vein as two parallel veins, and (3) we did not independently verify the accuracy of the vein width parameter generated using phenoVein.

To assess potential differences in venation patterning along the proximal‐distal axis of leaf lamina (base, middle, or apex), we first applied a standard outlier analysis to exclude rare outliers in the data set that likely represent artifacts (Baker et al., 2018). Our analysis detected many outliers for areole area because the data for this parameter has a strong positive skew. Because the identified outliers were not rare, they likely represent biologically relevant data and were retained throughout the analyses. Instead, we performed a square‐root transformation to improve the normality of the distribution of the areole areas. All the analyzed data are available at https://github.com/rlbaker5/AppsInPlantSci_phenoVein.

For parameters with only a single data point per image (number of branch points, number of end points, areole density, and number of areoles), we constructed a full model with location as a fixed effect and plant nested within accession as a random effect using the lmerTest package in R (Kuznetsova et al., 2017). For the variable areole area, there were multiple observations within each image necessitating a slightly more complex full model, where location was treated as a fixed effect and the random effect was location nested within plant nested within accession (Crawley, 2012). For each parameter, we compared the full models with null models that did not include fixed effects using analysis of variance and Akaike information criterion (AIC) scores. Sample sizes precluded testing the effect of crop type. When there was no significant difference between models, we opted for the simplest model. When there was a significant difference between models, we selected the model with the lowest AIC score.

## Results

### Tissue preparation and image acquisition

**Figure 1:**
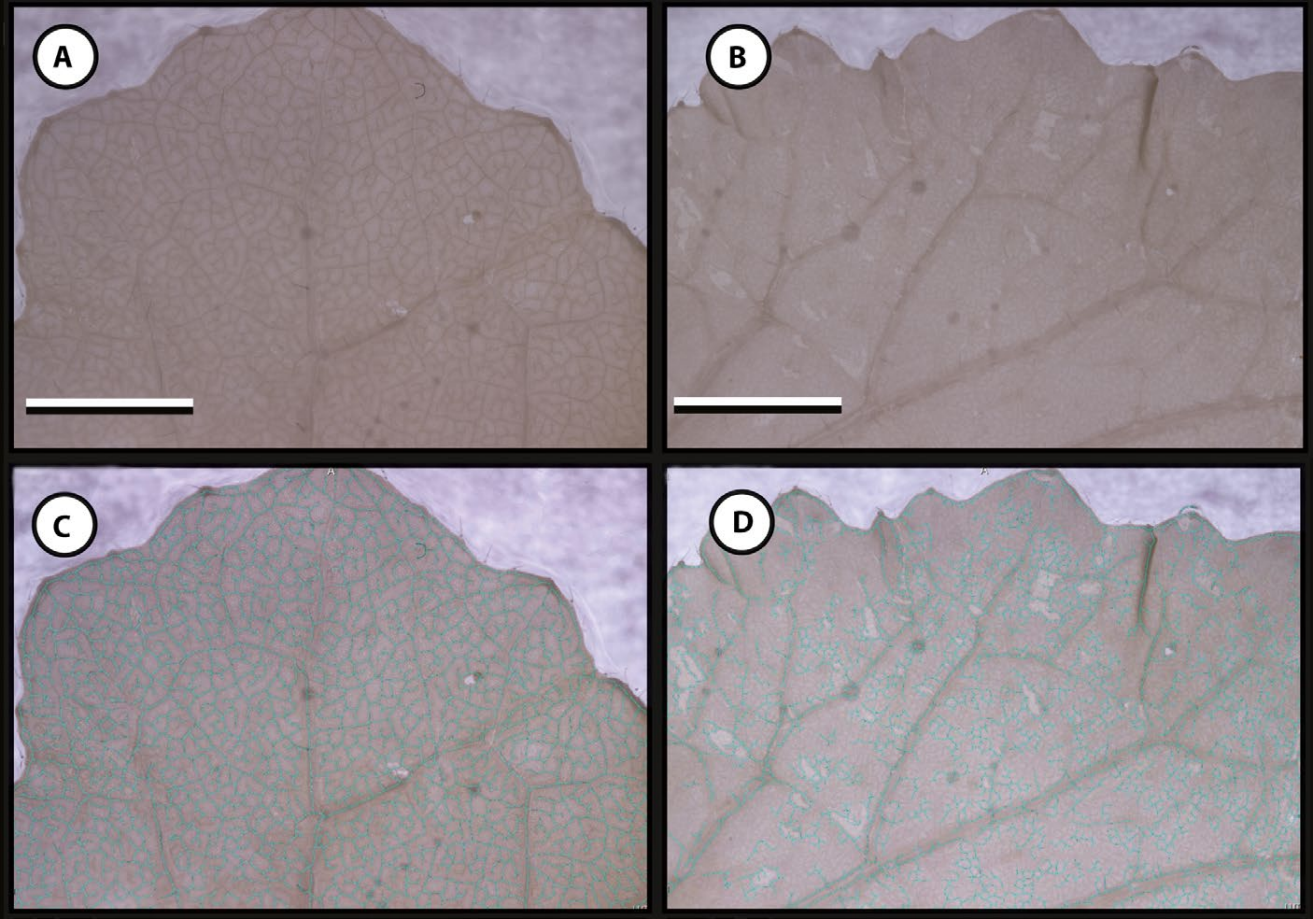
Images of *Brassica rapa* leaves acquired with a digital SLR camera and macro lens from the middle of two laminae. In each image, the midvein is below and out of the field of view. (A) A leaf that resulted in a high‐quality, high‐contrast image. (B) A damaged leaf resulting in a poor image with low contrast. (C) phenoVein was able to estimate the venation network for the high‐quality image in (A) and few manual corrections were necessary. (D) phenoVein was unable to estimate the venation network for low‐quality images such as in (B); the extensive manual corrections necessary preclude efficient data generation. Scale bars = 5 mm.

**Figure 2:**
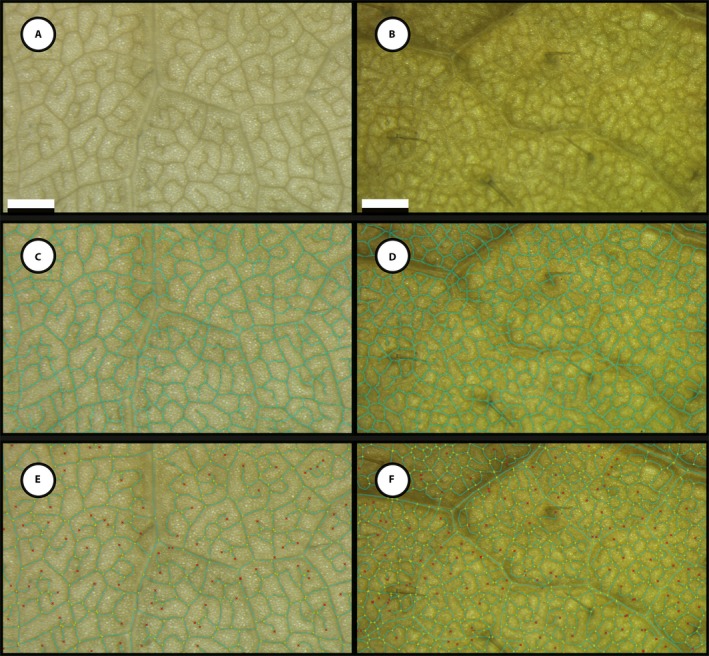
Images from the same *Brassica rapa* leaves and location as in Fig. 1 acquired via a stereomicroscope at 10× magnification. (A) As in Fig. 1A, a high‐quality, high‐contrast image was obtained. (B) Using a stereomicroscope results in a higher‐quality image than in Fig. 1B. (C) phenoVein estimates the venation skeleton from the high‐quality image in (A) very well. (D) Although phenoVein also estimates the venation skeleton for the image in (B), there were noticeable areas that required manual correction, particularly surrounding larger‐diameter veins. (E, F) Images after manual correction (compare C with E and D with F) of the estimated venation skeleton. Vein endpoints (red) and branch points (yellow) are identified. Scale bars are 1 mm.

We asked whether a standard tissue fixation protocol (FAA followed by 70% ethanol for storage) was sufficient for generating leaf venation data without employing specific clearing and staining procedures. After fixation, we trialed two methods of image acquisition. First, we used a digital SLR camera with a macro lens following Bühler et al. (2015) (Fig. 1A, B). Although the macro lens produced images with sufficient contrast between the veins and mesophyll tissue for phenoVein to estimate reasonable vein skeletons for some leaves (Fig. 1A, C; backgrounds have been removed to delete text that is automatically generated by phenoVein), for other leaves these images were not of sufficient quality for phenoVein to estimate a reasonable venation skeleton (Fig. 1B, D). When these same leaves as in Fig. 1 were imaged using a stereomicroscope and transillumination, they resulted in high‐contrast images (Fig. 2A, B) with venation skeletons that were readily detected by phenoVein (Fig. 2C, D). We therefore present results exclusively from the stereomicroscope‐acquired images.

### phenoVein workflow

After leaf collection and image acquisition, the entire phenoVein workflow took between 30 and 90 minutes per image. This time includes 15–20 minutes of computational time and 10 (Fig. 2A, C, E) to 60 (Fig. 2B, D, F) minutes of manual adjustments to the venation network estimated by phenoVein. High‐quality images with distinct contrast between the veins and parenchyma (e.g., Fig. 2A) had well‐estimated venation networks (Fig. 2C), requiring little manual adjustment (Fig. 2E). Images acquired from thicker laminae had less contrast between the parenchyma and veins (Fig. 2B), likely because of the increased number of cell walls and increased retention of plastids after their fixation and ethanol washes. However, even for relatively low‐contrast images acquired using a stereomicroscope, phenoVein estimated reasonable venation skeletons (Fig. 2D), which required relatively little manual correction (compare Fig. 2D, F). An enlarged inset example of manual corrections is presented in Appendix S1.

### Data management using custom R scripts

Formatting data for analysis can be a time‐consuming procedure and, if done manually, can also introduce unnecessary errors. phenoVein generates a number of output files for each image, including a .csv file containing the data from the estimated vein network; however, these data must be combined and reformatted prior to data analysis. We generated an annotated R script (available with example .csv output files from phenoVein at https://github.com/rlbaker5/AppsInPlantSci_phenoVein) to automate this process. Our script accesses all .csv output files (potentially from multiple images) that are kept in a single user‐specified folder. It extracts the data, reshapes it into a “rectangular” format to facilitate statistical analyses, compiles all the data from all the images into a single data set, and writes a new .csv file to the user's machine that can be imported into, and easily analyzed with, common statistical software packages.

### The effect of location

To assess the effect of location (base, middle, or apex) along the leaf lamina on aspects of leaf venation, we employed a series of mixed‐effects linear models. Our full models consisted of both fixed (location) and nested random (plant identity within accession) effects to account for potential pseudoreplication. In all cases, the full model was significantly better than the reduced models containing only random effects, indicating that there was a significant effect of location on all dependent variables. We therefore present the relevant *F* values, degrees of freedom, and associated *P* values for the fixed effect of location from the best model (Table 2). The leaf apices had fewer end points, fewer branch points, fewer areoles, a shorter skeleton length, lower vein density, and larger areole areas than the base or middle of the leaf (Appendix S2).

**Table 2:** Linear mixed‐effects models testing the effect of location (base, middle, or apex) on aspects of leaf venation. Location was treated as a fixed effect and plant replicate nested within accession ID was treated as a random effect. Areole area had an additional level of nesting where location was nested within plant and accession ID.

Trait	*F* value_(df)_	*P* value
End points	*F*_(2, 39.46)_ = 3.819	0.03046
Branch points	*F*_(2, 41.23)_ = 6.495	0.00353
Areole number	*F*_(2, 42.20)_ = 5.273	0.009141
Skeleton length	*F*_(2, 41.02)_ = 3.227	0.04993
Vein density	*F*_(2,41.14)_ = 7.601	0.001553
Areole area	*F*_(2,40.03)_ = 21.435	4.698e–07

## Discussion

phenoVein is an effective method of quantifying leaf venation in *Brassica rapa*. Leaves fixed in FAA and stored in ethanol require no additional clearing or staining, and image acquisition requires minimal specialized equipment. Our sample preparation protocol avoids difficult‐to‐obtain tissue clearing reagents such as Rotisol (Carl Roth, Karlsruhe, Germany), which is unavailable in the United States, or chloral hydrate, which requires permission from state pharmaceutical boards to purchase. Additionally, eliminating clearing and staining steps increases sample throughput by avoiding the multi‐day protocols necessary for using alternative clearing reagents such as clearSee (Kurihara et al., 2015).

Sample throughput depends on image quality. Although Bühler et al. (2015) used a digital SLR camera and a macro lens for image acquisition, we found that our SLR camera and macro lens did not always result in images of sufficient quality for venation detection via phenoVein. Several factors contributed to our inability to use a digital SLR for all leaves: we had a lower‐quality lens and our camera did not have a full‐frame digital sensor. Additionally, our leaves were larger, older, and we did not clear or stain them. We found that the stereomicroscopes with transmitted light were able to capture images of sufficient quality for analysis in phenoVein from all leaves without staining or clearing, even when a digital SLR could not. In addition to the increased quality of lens, camera, and light source compared with the digital SLR, the stereomicroscope had a decreased field of view, which allowed us to avoid damaged areas, folds caused by the three‐dimensionality of leaf laminar growth, and problematic larger veins. Our tissue preparation protocol worked well across all three *B. rapa* crop types spanning four different subspecies. Preliminary testing in our lab indicates that this protocol also works well in several closely related and economically important *Brassica* species (*B. carinata* A. Braun, *B. juncea* (L.) Czern., *B. oleracea* L., *B. nigra* W. D. J. Koch, and *B. napus* Vilm.). While we do not surmise that this protocol will be successful for very thick leaves, we anticipate it is likely to succeed across a broad array of species with similar leaf thicknesses.

Once sufficient‐quality images were acquired, we used phenoVein to detect patterns of leaf venation. phenoVein is freely available, has a user‐friendly graphical interface, and can be run on a standard desktop computer. The semi‐automated leaf quantification feature and comprehensive workflow make phenoVein a tool that undergraduate student researchers are capable of using. In particular, the ability to manually edit venation skeletons facilitates data acquisition from suboptimal images (compare Figs. 2D and 2E) and contributed to our ability to skip leaf clearing and staining.

Using this new method, we found that leaf laminae are not uniform across their proximal‐distal axis. Previous studies have documented variation in aspects of physiology, including stomatal conductance and photosystem II efficiency, and micromorphological characters such as stomatal density and size (Wang and Clarke, 1993; Weyers and Lawson, 1997; Enríquez et al., 2002; Nardini et al., 2008; Ocheltree et al., 2012). Despite the fact that these functional aspects of leaves could plausibly be related to leaf venation, few studies have examined intralaminar variation in venation. The studies that do quantify venation tend to be restricted to very large leaves and revealed no significant differences within the laminae (Li et al., 2013; Li and Cao, 2014). One exception was a study of tobacco, which showed that vein densities are greatest at the apex of the leaf lamina (Nardini et al., 2008). In contrast, we found that the location along the proximal‐distal axis of the *B. rapa* leaf laminae significantly affected all aspects of leaf venation (Table 2), with leaf apices generally having fewer end points, fewer branch points, fewer areoles, a shorter skeleton length, lower vein density, and larger areole areas than the base or middle of the leaf (Appendix S2). This contradiction with the results of the previously published studies may be an artifact of different data collection techniques, may reflect the phylogenetic signal (where monocots, even those without parallel venation, are less likely to exhibit proximal‐distal variation), or may indicate patterns of selection. Linking the intralaminar venation variation to functional micromorphology or physiology such as stomatal characteristics or photosynthetic rates could bolster the selection hypothesis. The potential association between intralaminar venation variation and functional traits remains an intriguing question.

With the increased pressure on freshwater resources due to climate change and population growth, understanding the connections between plant physiology and venation could yield important avenues for crop improvement. However, quantifying leaf venation has historically been time consuming and labor intensive. Here, we demonstrate that minimal protocols for leaf preparation can be used to generate leaf venation data in sufficient quantities for robust statistical analyses using the semi‐automated phenoVein program. We provide an R script to facilitate the downstream analysis of phenoVein data. These protocols are fast, easy to use, and require little specialized equipment. Taken together, they have the potential to facilitate our understanding of leaf venation and the connections between venation and physiology, and ultimately to improve crop yields.

## Author Contributions

R.L.B. conceived of the research questions, grew the plants, collected tissue, and performed the data analysis and interpretation. E.L.N., G.L.B., and J.L. trialed the clearing, staining, and image acquisition protocols and collected the venation data. R.L.B., E.L.N., and G.L.B. drafted and revised the manuscript. E.L.N., G.L.B., J.L., and R.L.B. give their final approval for publication and agree to be accountable for all aspects of the work.

## Supporting information


**APPENDIX S1.** An example of manual corrections. (A) Panel A from Fig. 2 with a black inset box indicating the area of enlargement in panels B–D. (B) An enlarged view of the inset area in (A). (C) An enlarged view of the inset area in (A) with automatically generated veins. (D) An enlarged view of the inset area in (A) after manual correction. Vein endpoints (red) and branch points (yellow) are identified. Scale bar = 1 mm.

**APPENDIX S2.** Box plots summarizing the values at the base, middle, and apex of leaves for all the data analyzed.

## Data Availability

All data and products are freely available at https://github.com/rlbaker5/AppsInPlantSci_phenoVein.
