# Supplementary material for: Variation within laminae: Semi‐automated methods for quantifying leaf venation using phenoVein
Source: Appl Plant Sci. 2020 May 11;8(5):e11346. doi: 10.1002/aps3.11346 (PMC7249269; doi:10.1002/aps3.11346)

DOI

**APPENDIX S1.** An example of manual corrections. (A) Panel A from Fig. 2 with a black inset box indicating the area of enlargement in panels B–D. (B) An enlarged view of the inset area in (A). (C) An enlarged view of the inset area in (A) with automatically generated veins. (D) An enlarged view of the inset area in (A) after manual correction. Vein endpoints (red) and branch points (yellow) are identified. Scale bar = 1 mm.

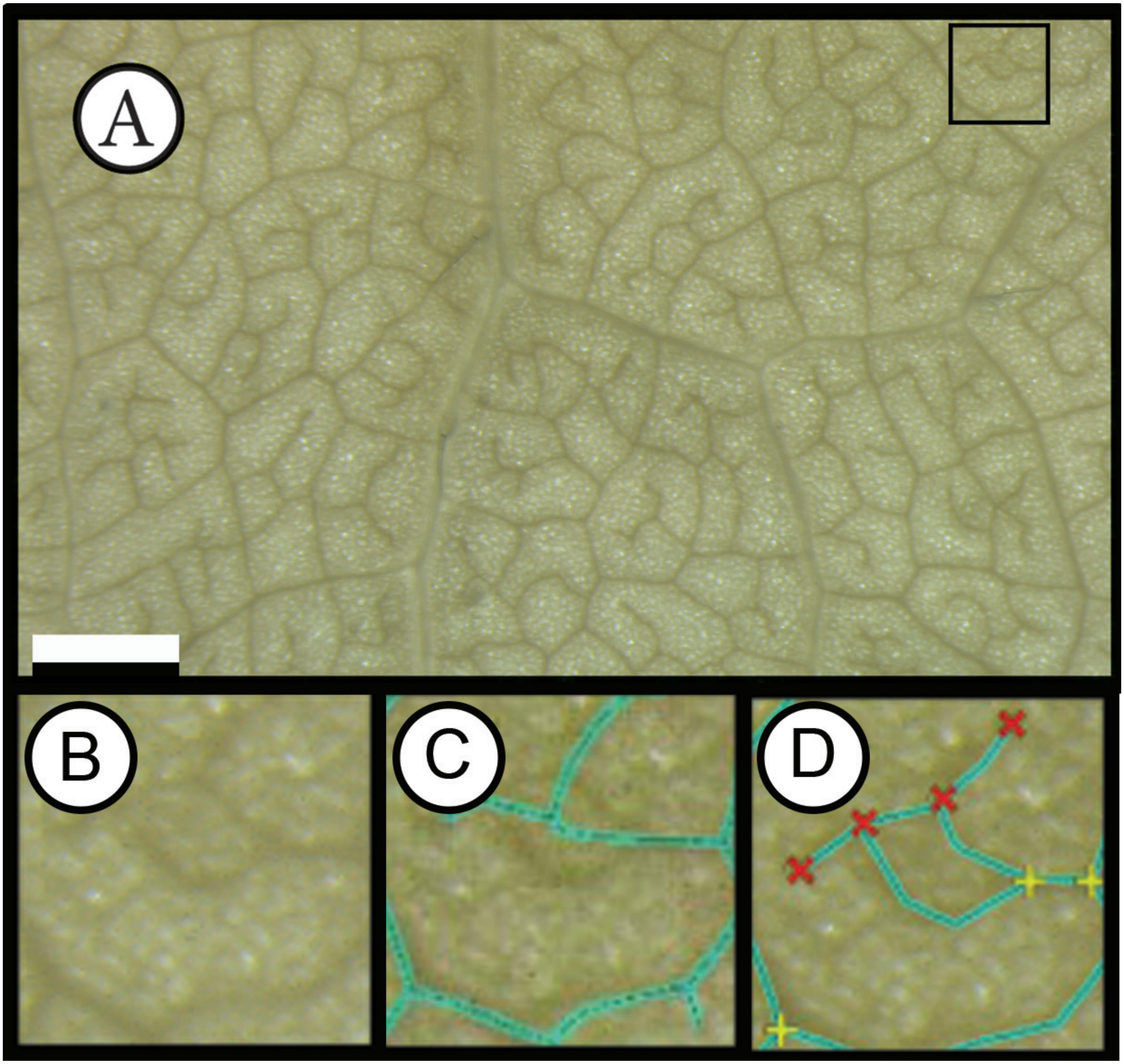

Supplement: Supplementary file 1 — APPENDIX S1. An example of manual corrections. (A) Panel A from Fig. 2 with a black inset box indicating the area of enlargement in panels B–D. (B) An enlarged view of the inset area in (A). (C) An enlarged view of the inset area in (A) with automatically generated veins. (D) An enlarged view of the inset area in (A) after manual correction. Vein endpoints (red) and branch points (yellow) are identified. Scale bar = 1 mm. [file APS3-8-e11346-s001.pdf]
