# Supplementary material for: Variation within laminae: Semi‐automated methods for quantifying leaf venation using phenoVein
Source: Appl Plant Sci. 2020 May 11;8(5):e11346. doi: 10.1002/aps3.11346 (PMC7249269; doi:10.1002/aps3.11346)
Supplement: Supplementary file 2 — APPENDIX S2. Box plots summarizing the values at the base, middle, and apex of leaves for all the data analyzed. [file APS3-8-e11346-s002.pdf]

DOI

APPENDIX S2. Box plots summarizing the values at the base, middle, and apex of leaves for all the data analyzed.

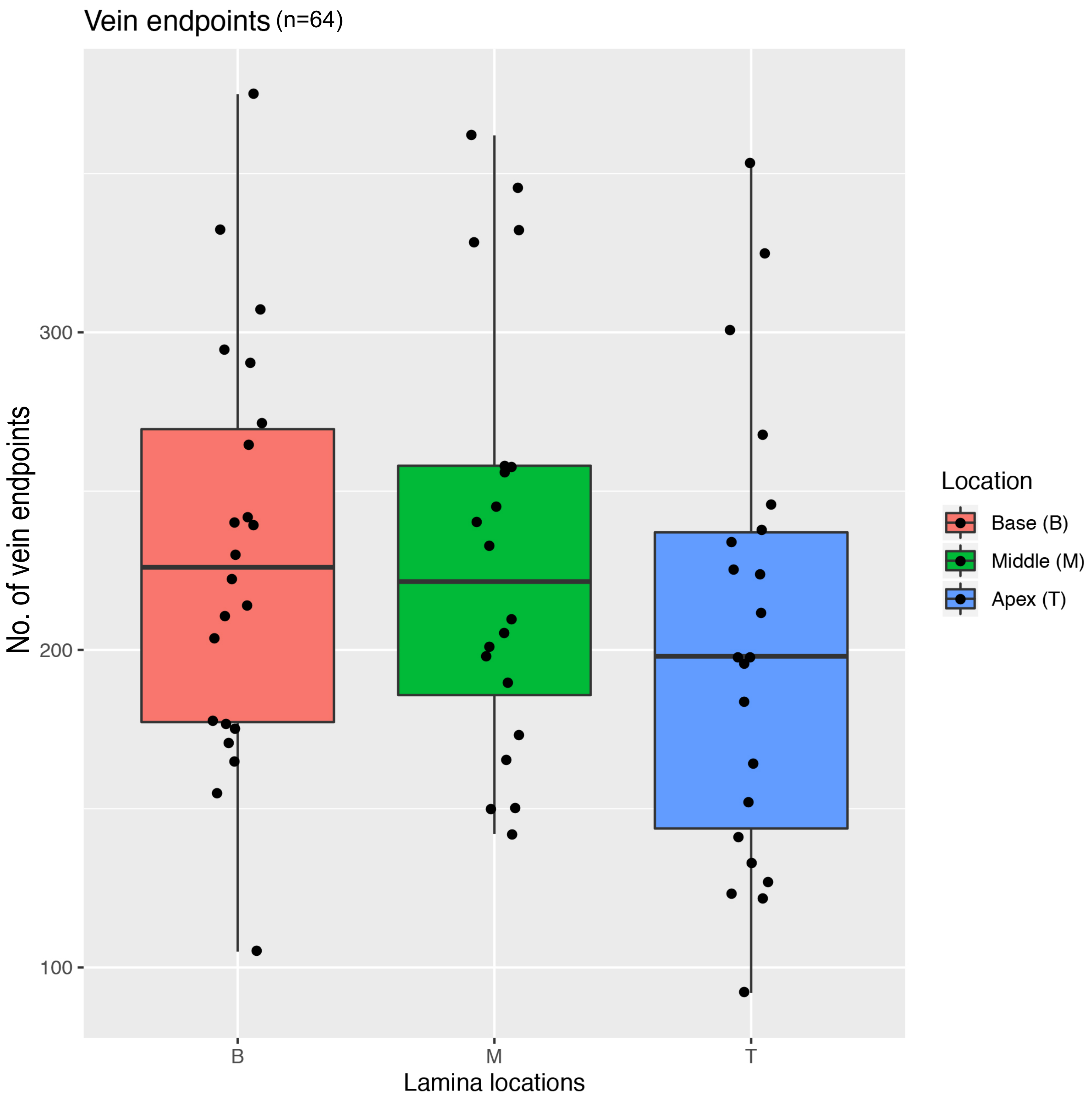

DOI

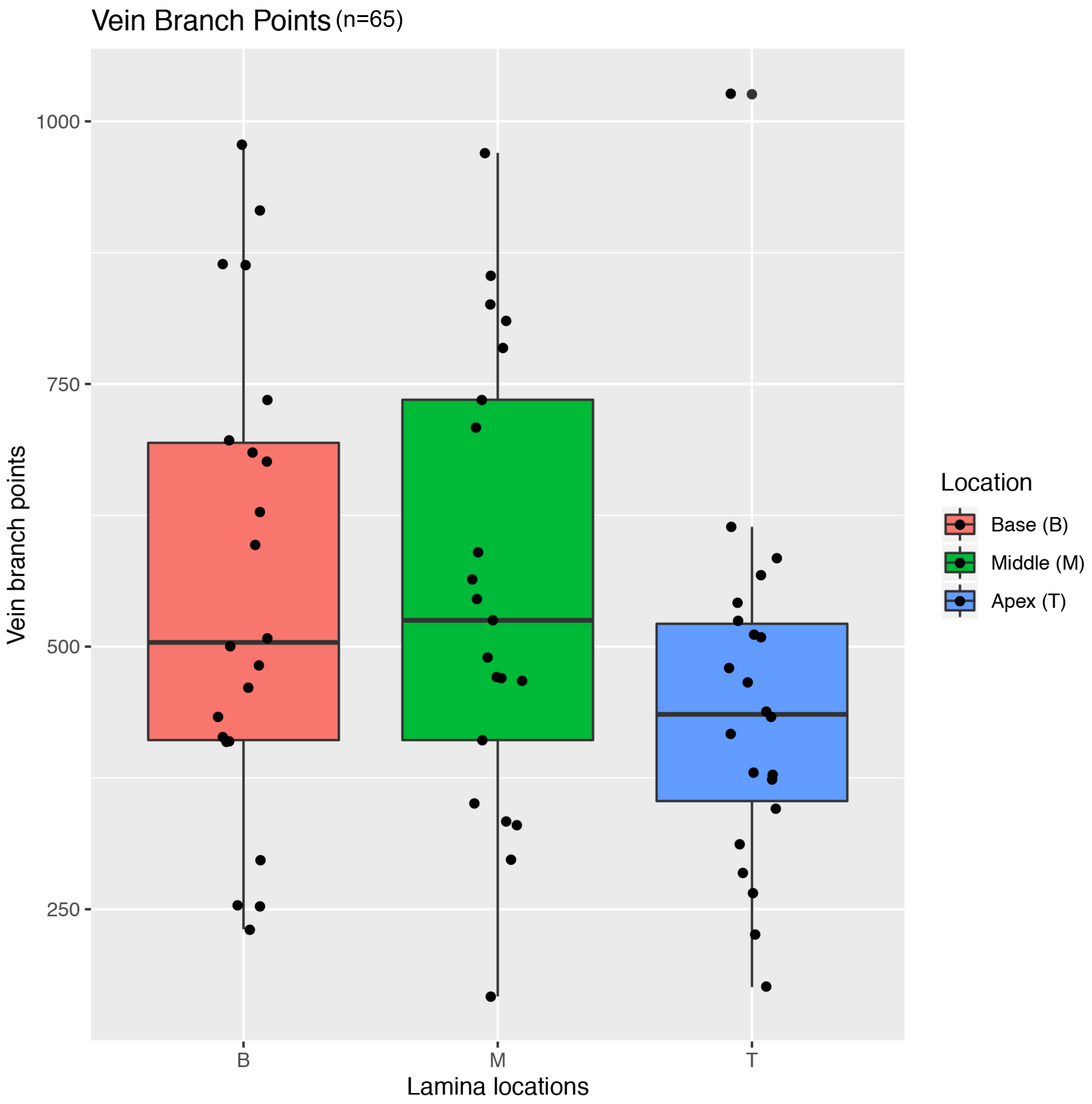
Vein Branch Points (n=65)
Vein branch points
1000
750
500
250
B
M
T
Lamina locations
Location
Base (B)
Middle (M)
Apex (T)

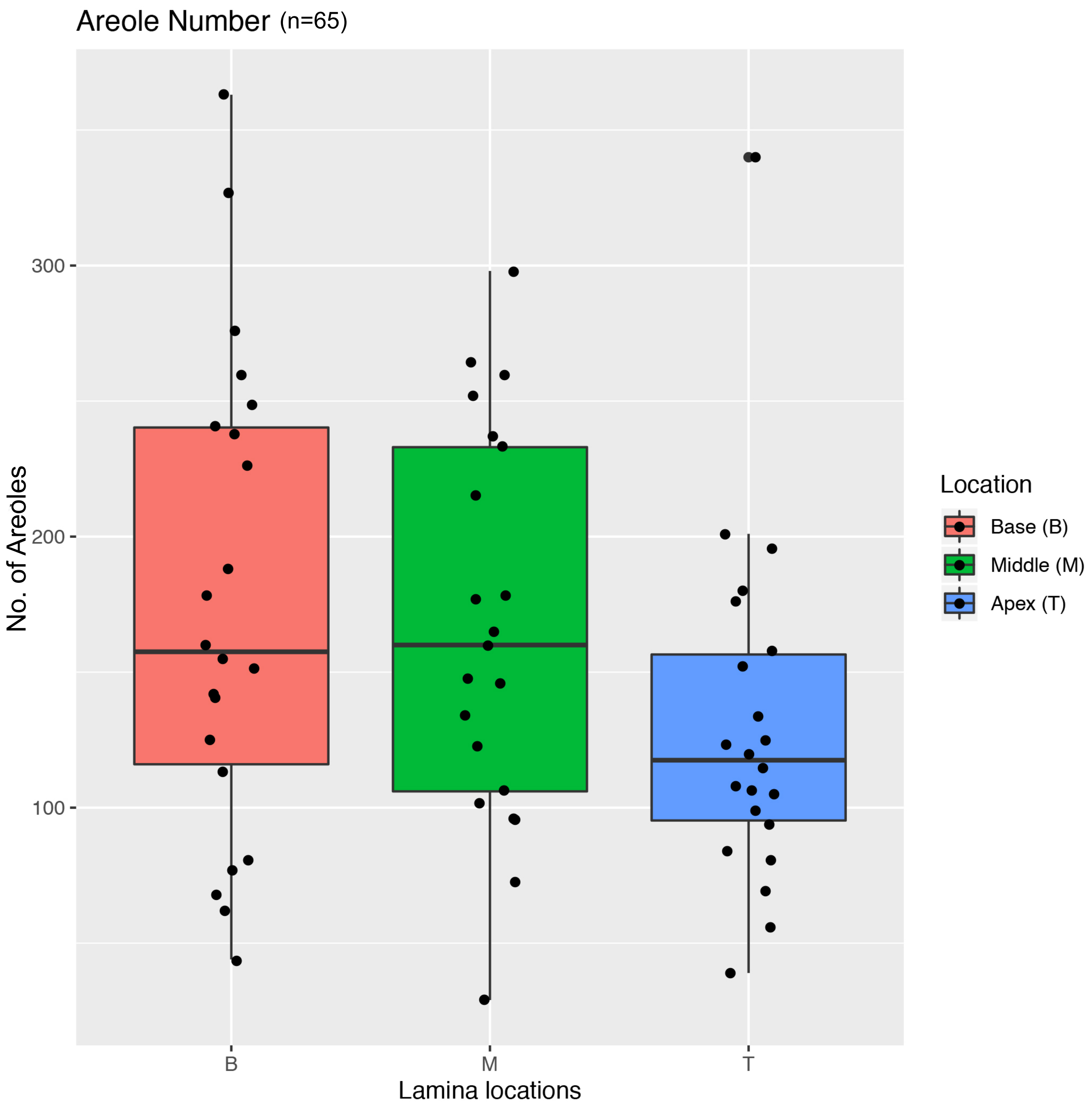
Areole Number (n=65)
No. of Areoles
300
200
100
B
M
T
Lamina locations
Location
Base (B)
Middle (M)
Apex (T)

DOI

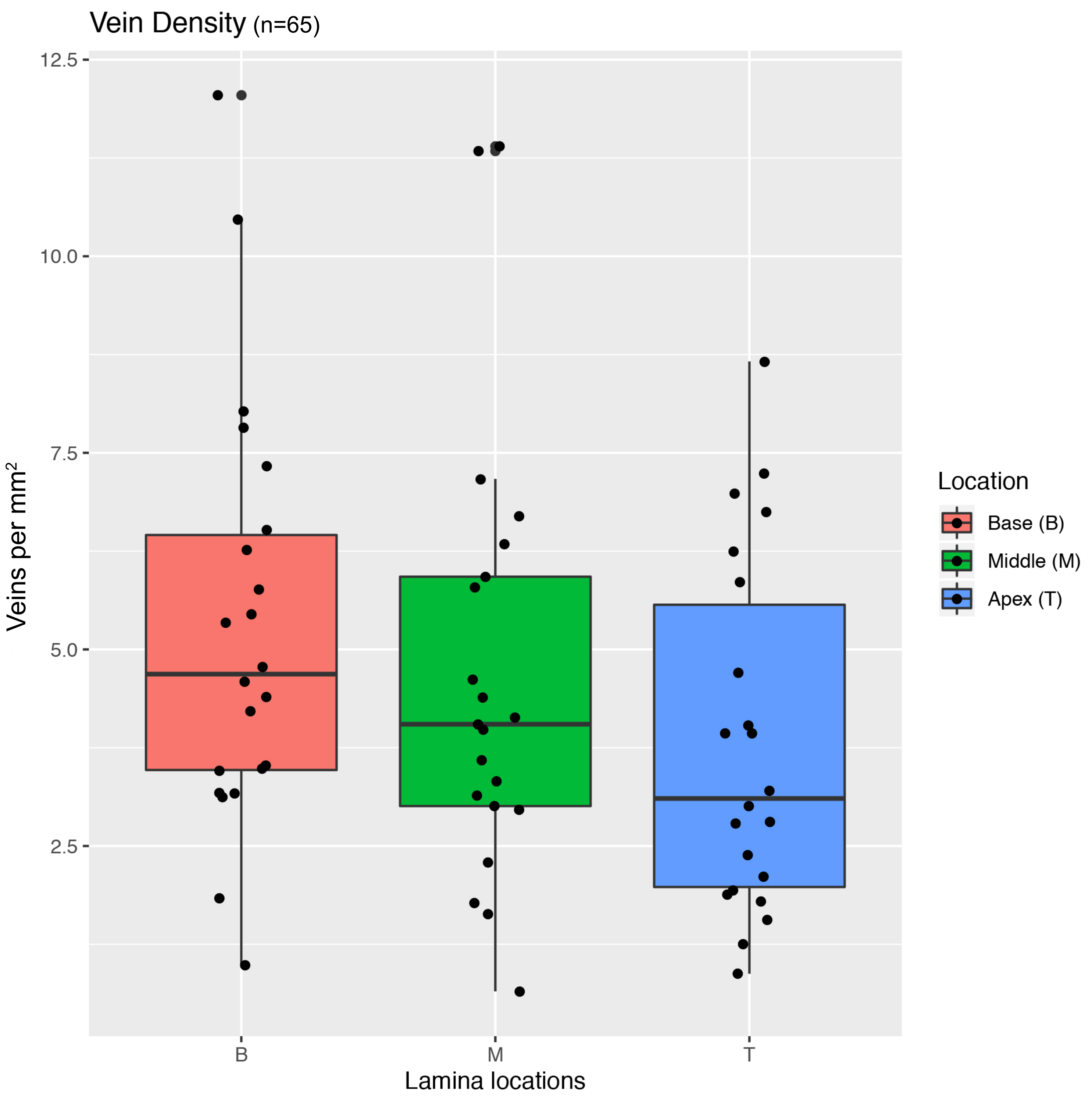
Vein Density (n=65)
12.5
10.0
7.5
5.0
2.5
Veins per mm²
B
M
T
Lamina locations
Location
Base (B)
Middle (M)
Apex (T)

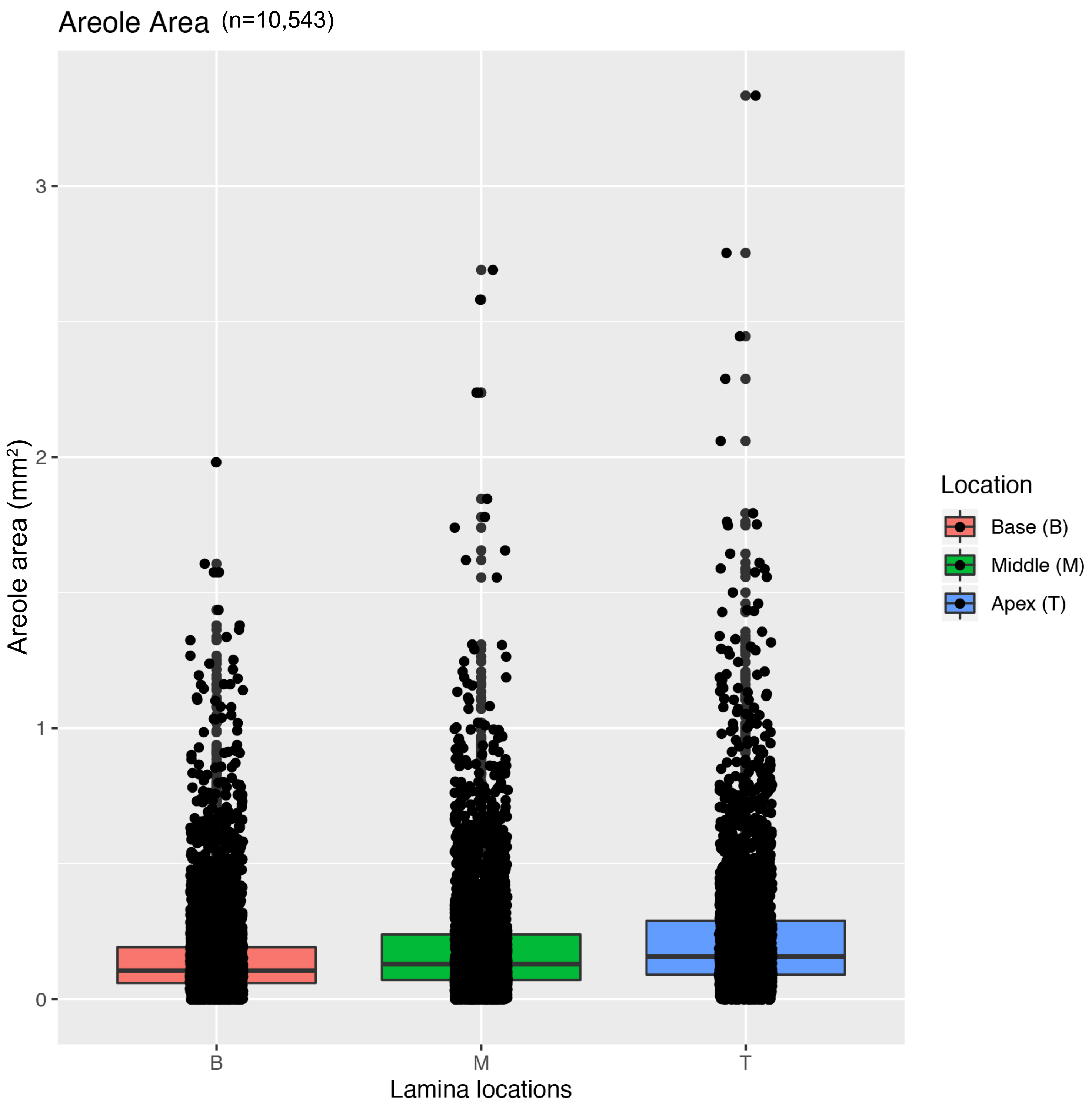
Areole Area (n=10,543)
Areole area (mm²)
0
1
2
3
B
M
T
Lamina locations
Location
Base (B)
Middle (M)
Apex (T)
